# Ion Channels and Transporters as Therapeutic Agents: From Biomolecules to Supramolecular Medicinal Chemistry

**DOI:** 10.3390/biomedicines10040885

**Published:** 2022-04-12

**Authors:** Giacomo Picci, Silvia Marchesan, Claudia Caltagirone

**Affiliations:** 1 Chemical and Geological Sciences Department, University of Cagliari, 09042 Cagliari, Italy; gpicci@unica.it; 2 Chemical and Pharmaceutical Sciences Department, University of Trieste, 34127 Trieste, Italy

**Keywords:** ion channels, ion transporters, ionophores, ion carriers, channelopathies, cystic fibrosis, AMPs, peptides, supramolecular medicinal chemistry, nanotubes

## Abstract

Ion channels and transporters typically consist of biomolecules that play key roles in a large variety of physiological and pathological processes. Traditional therapies include many ion-channel blockers, and some activators, although the exact biochemical pathways and mechanisms that regulate ion homeostasis are yet to be fully elucidated. An emerging area of research with great innovative potential in biomedicine pertains the design and development of synthetic ion channels and transporters, which may provide unexplored therapeutic opportunities. However, most studies in this challenging and multidisciplinary area are still at a fundamental level. In this review, we discuss the progress that has been made over the last five years on ion channels and transporters, touching upon biomolecules and synthetic supramolecules that are relevant to biological use. We conclude with the identification of therapeutic opportunities for future exploration.

## 1. Introduction

### 1.1. Ion Channels and Ion Transporters as Therapeutic Targets

**Figure 1:**
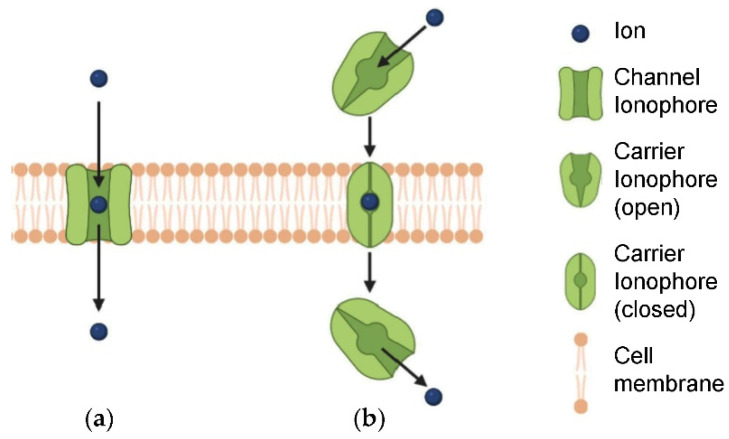
(**a**) Ion channels and (**b**) ion transporters. Reproduced with permission from [26]. Copyright Elsevier 2022.

Natural ion channels are composed of large proteins that form pores spanning through lipid membranes (Figure 1a), and they can be grouped based on ion selectivity (e.g., Na^+^, Cl^−^) [1], and type of gating mechanism (e.g., activation mediated by voltage [2], ligand [3], mechanical [4,5], or light stimuli [6]). They are involved in a plethora of physiological and pathological processes, for which they have been attracting increasing interest for therapeutic intervention [7]. Remarkably, 19% of drugs that have been approved by the U.S. Food and Drug Administration (FDA) target ion-channel proteins, both gate-activated and ligand-activated [8]. Research in ion-channel modulation has become a hot topic, and encompasses both synthetic molecules [9] and biomolecules, such as lipids [10,11,12], toxins [13], antibodies and nanobodies [14], and venom-derived peptides [15,16]. Dysfunction of ion channels is linked to several pathologies that are generally termed channelopathies, which comprise a plethora of diverse diseases, of the central nervous system, the renal system, and cardiac tissue, as recently reviewed [17], and many others, affecting for instance the skeletal muscle [18], the immune response [19], and glucose levels [20]. The reasons for channel dysfunction are diverse, and include mutations [21,22,23] and post-translational defects [24,25].

Ion transporters are considered a separate class that plays a role in the regulation of ion homeostasis. They are mobile and they can travel through cell membranes. They transiently bind ions to enable their crossing through the lipid barrier (Figure 1b). They have been proposed as anticancer agents and sensitizers, and they include both natural biomolecules (e.g., peptides and antibiotics) and synthetic molecules (e.g., polyethers, crown ethers) [27,28]. They have been proposed as antimicrobial (AM) agents [26,29,30,31], with the added benefit of immunomodulation activity [26]. However, their successful use in therapy suffers from unsolved challenges in formulation, and in targeting, thus resulting in sub-optimal performance and side-effects. A potential solution could be their selective activation at the pathological site [28]. 

In therapy, channel blockers are used to treat numerous diseases. As a well-known example, selective Ca^2+^-channel blockers are anti-hypertensive drugs [32]. Several types of ion transporters are being considered as therapeutic targets for ischemic stroke [33]. They include transient receptor potential (TRP) channels, which are an inhibition target to treat not only stroke, but also depression, epilepsy, and neurodegenerative forms [34]. In fact, Ca^2+^-channel blockers used to treat hypertension were found to exert a neuroprotective effect against Alzheimer’s disease and depression [35]. Dysregulation of Ca^2+^ homeostasis was found to occur ubiquitously in Alzheimer’s disease, and thus it has been proposed as a therapeutic target for neurodegeneration [36,37].

Na^+^ and Ca^2+^ channel blockers find use in the treatment of bipolar disorders too, and another ion-related therapy that is effective for some patients includes administration of lithium salts [38]. Ca^2+^ channel blockers attenuate cellular hyperactivity, and for this reason they are considered in psychiatry to treat various disorders [39]. Na-K-Cl cotransporters are therapeutic targets for many diseases, including pain, epilepsy, brain edema, and hypertension [40]. Furthermore, Ca^2+^ and Na^+^ channel blockers are used to treat chronic and neurogenic pain [41]. Acid-sensing ion channels have been proposed as therapeutic target for migraine [42].

Recently, however, disruption of ion channel function was found to play a role in the etiology of type-2 diabetes [43]. Several types of ion channels are dysregulated in diabetes and could serve as therapeutic targets, although a key limitation is the risk of side effects arising from sub-optimal selectivity of channel blockers [44]. Furthermore, insulin resistance and altered Ca^2+^ homeostasis are associated with non-alcoholic liver steatosis, which is another important pathology that would benefit from Ca^2+^-channel targeting, as long as it is specific for pathologically relevant channel isoforms that are expressed in the liver [45]. 

**Figure 2:**
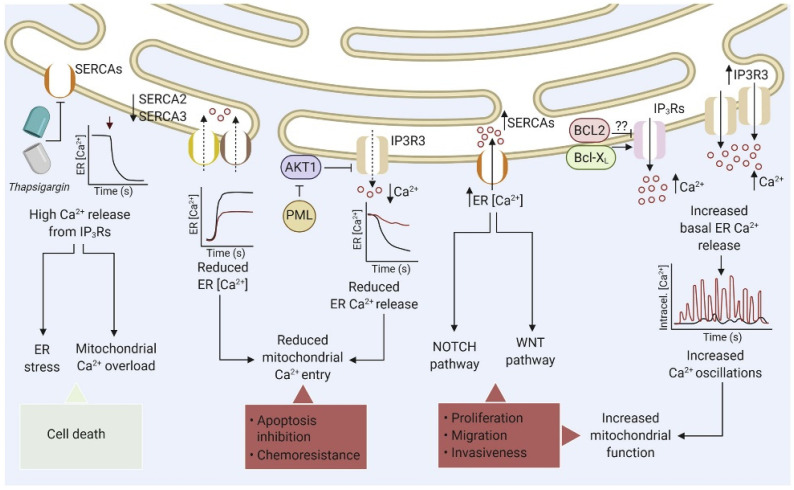
Cancer-associated defects of endoplasmic reticulum Ca^2+^ homeostasis. Reproduced with permission from ref. [51]. Copyright Elsevier 2020.

Overall, selectivity is indeed key, and there is a recent trend to employ biologics to achieve it, including engineered antibodies, nanobodies, and venom-derived peptides [46]. Besides organ-specificity (e.g., cardiac [47], cerebellar [48], and liver [45] tissues), organelle-specificity is also relevant, for instance to target ion channels of mitochondria [49] or lysosomes [50]. In particular, mitochondrial-associated endoplasmic reticulum membranes (MAMs) contain specific proteins and ion channels that control key cellular processes, such as redox homeostasis and Ca^2+^ signaling, and their alteration has been linked to several pathologies, including cancer (Figure 2) [51]. Likewise, lysosomes are underestimated regulators of Ca^2+^ levels that could be interesting targets for anticancer agents [51]. Several ion-channel blockers are being considered for the treatment of cancer metastases (Table 1), which are accompanied by ion alterations and are characterized by unique protein expression patterns that are different from the originating tumors and target host tissue [52].

Detailed knowledge of specific expression of the various types of ion channels is thus of paramount importance to develop effective therapy and reduce side-effects [53]. Alternatively, organism selectivity could be exploited to develop antiviral agents targeting ion channels (i.e., viroporins), which play key roles in the virus lifecycle and are immunomodulatory [54].

Besides cationophores, anionophores could also be interesting targets for therapeutic treatment. In particular, Cl^−^ ion transporters are relatively underexplored to treat various diseases, such as constipation and secretory diarrheas, kidney stones, and polycystic kidney disease, but also dry eye disorders, hypertension, and even osteoporosis [55].

### 1.2. Therapeutic Activation of Ion Channels and Ion Transporters

**Figure 3:**
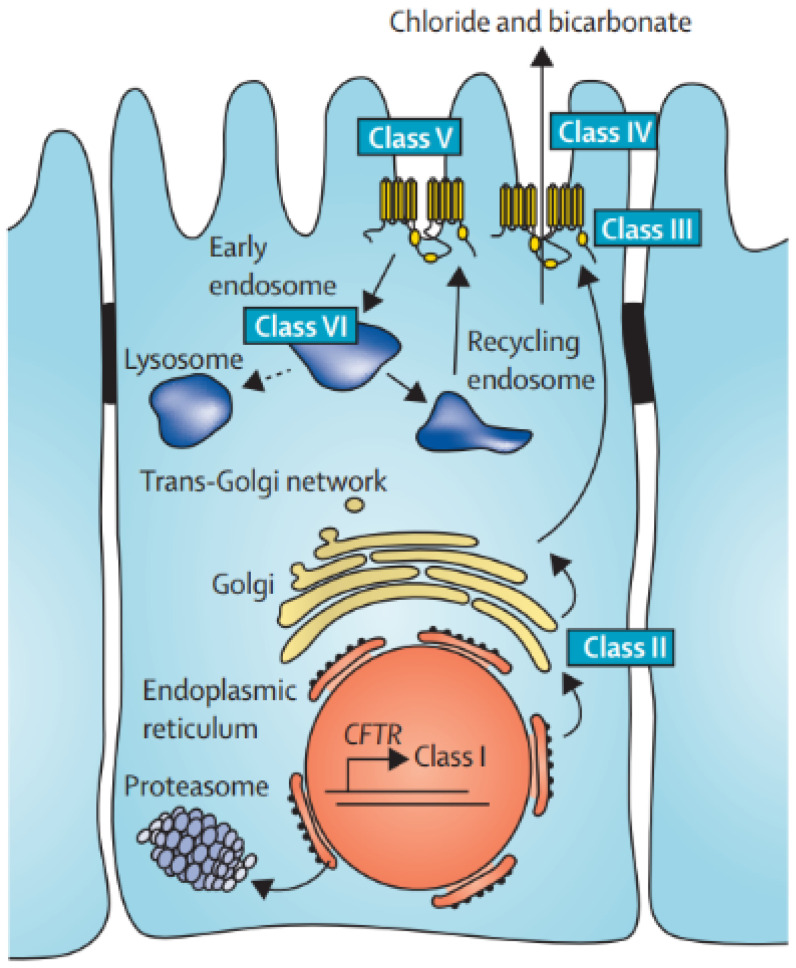
Cystic fibrosis (CF) arises from defective anion channels on epithelial cells, due to *CFTR* mutations that are grouped into several classes, depending on the cellular process that results impaired. Reproduced with permission from [59]. Copyright Elsevier 2021.

The pathology that is mostly known for the therapeutic effects of ion-channel activation is cystic fibrosis (CF). This disease has been ascribed to mutations of the CF transmembrane conductance regulator (*CFTR*) gene (Figure 3), which encodes for the epithelial anionic channel that transports Cl^−^ and HCO_3_^−^, resulting in defective mucus hydration and clearance. The resulting clinical manifestations are diverse and multi-organ, affecting especially the lungs, and the gastrointestinal and the endocrine systems. Management strategies have been traditionally aimed at treating symptoms, while interventions at the root of the problem through modulation of ion-channel activity have just opened a whole new scenario for improved life-quality of affected patients. In particular, the identification of mutations in *CFTR* in CF patients has enabled the development of targeted therapies to restore the function of the ion channel [56]. Besides Cl^−^ and HCO_3_^−^ channel activators [57], several biologics are used to restore the channel function, i.e., gene therapy and editing, RNA therapy and micro RNAs [58]. Readers interested in further details pertaining this pathology and treatment strategies are referred to an excellent and comprehensive recent review [59]. 

The therapeutic potential of ion channel activation goes well beyond CF and represents an unexplored avenue for new therapies. For instance, potentiation of specific sub-types of TRP channels has been proposed to prevent and treat obesity [60]. Importantly, dysregulation of Na^+^, K^+^, Ca^2+^, or Cl^−^ intracellular levels can lead to programmed cell death, and for this reason several ion-channel modulators have been proposed against cancer [61]. It is worth noting that increasing cytosolic Ca^2+^ concentration can have opposite downstream effects in cancer cells, depending on the biochemical activation pathway [62]. Therefore, not only channel blockers, but also activators or artificial channels could pave the way to new therapeutic approaches, as long as cancer cell selectivity is achieved. 

Restoration of cation transport is another underexplored avenue that can be of therapeutic relevance. For instance, impaired Na^+^ transport was recently identified as an upstream pathogenic factor in inflammatory bowel disease [63]. Numerous enteric pathogens target the expression and/or function of ion transporters, causing diarrhea [64]. Another type of cation transport that is neglected pertains Mg^++^ channels. Mg^++^ homeostasis is regulated by several ion channels that are either downregulated or upregulated in many digestive cancers, thus providing an opportunity for innovative treatments [65]. Zn^++^ transporters were also found to ameliorate oxidative stress and insulin resistance, and were thus identified as new therapeutic targets [66].

## 2. Biomolecular Ion Channels and Transporters as Therapeutic Agents

### 2.1. Ion-Channel Biomolecules as Therapeutic Agents

There are several ion-channel biomolecules that have attracted interest not only for their therapeutic potential, but also as models to design artificial channels. The majority consist of polypeptides or proteins. They have enabled the development of synthetic 3-in-1 transporters for cations, anions, and zwitterions [67]. Here we will provide a brief overview of the most studied channels.

Ion-transporting rhodopsins are fascinating light-activated proteins that were firstly identified in photo-responsive chemotactic bacteria. Nowadays, they are mainly used in optogenetics, and they have inspired the design of biomimetics. The two main groups are light-driven ion-pumps, and light-gated ion channels. The former use light energy to transport specific ions (H^+^, Na^+^, Cl^−^) against electrophysiological potential, while the latter are less specific and employ passive transport. Light-activation of rhodopsin pumps results in hyperpolarization of membrane potential that effectively inhibits neuron firing, thus exerting an inhibitory role [68]. Many variants have been described, and the detailed mechanisms of their activity were recently reviewed [69,70]. Their therapeutic application has been envisaged mainly in gene therapy to restore vision in patients with degenerative retinal diseases, which otherwise can progress to blindness [71]. 

Natural KcsA K^+^ channels have inspired the biomimetic design of highly selective K^+^ transporters that require their dehydration to enable transport [72]. A columnar wire that alternates K^+^ ions and water molecules allows to overcome the electrostatic destabilization through the channel, and it was reproduced in supramolecular biomimetics [73]. Interestingly, inclusion of light-driven rotary molecular motors boosted ion transport, thanks to the continuous rotation that is beneficial to the mass-transport, offering a mechanism of action that is different to the common conformational switches [74].

Another interesting class of ion-channel therapeutics falls within the category of antimicrobial peptides (AMPs). They typically exert their bioactivity through the formation of pores in bacterial membranes, and some AMPs actually form ion channels. Of these, the most studied is gramicidin. Gramicidin A presents features alternating L- and D-amino acids, which increase proteolytic resistance and they enable the formation of an amphipathic transmembrane ion channel through dimerization. Several analogues are being continuously developed to reduce its hemolytic side-effects that limit systemic use [75]. Others are being optimized to induce apoptosis in cancer cells [76]. Gramicidin A has inspired the design of cation transporters [77], and recent biomimetics composed of helical foldamers could match gramicidin’s speed of proton transport [78].

The alternating D- and L-amino acid design is also effective to attain cyclopeptides that stack into nanotubular ion channels, as recently reviewed [79,80]. Interestingly, both homochiral [81,82] and heterochiral linear peptides as short as two amino acids can form supramolecular nanotubes that gel [83,84]. These systems could be interesting to develop smart AMs, so that the bioactive supramolecular channels are formed when needed [85], and presence of D-amino acids could improve AMPs’ pharmacokinetics [86].

Amongst non-peptidic natural ion channels, amphotericin B is a well-known antifungal agent produced by bacteria that forms non-selective channels, which are permeable to both cations and anions. In cultured cells derived from CF patients, it restored HCO_3_^−^ secretion, and, overall, host defenses. The process was independent from CFTR and it required functional interfacing with Na^+^, K^+^-ATPase [87]. This work offered an important proof of concept that biomolecule-based ion channels can compensate for deficient ion transport in human disease models, and it opened up new avenues for intervention [88].

### 2.2. Biomolecular Ion Transporters as Therapeutic Agents

There are several AMs that exert their activity through ion transport, as recently reviewed [26]. In many cases, the AM activity was well-known, and only later the ion transport was identified. Among peptides, daptomycin is one of the few AMPs that has been approved by the U.S. FDA for clinical use, with gramicidin, colistin, and vancomycin and its derivatives [89]. All of them derive from Gram-positive bacteria in the soil and disrupt lipid membrane organization. Daptomycin was recently found to bind Ca^2+^ and form transient ion carriers, leading to ion leakage [90]. Among the non-peptidic natural molecules, several polyethers act as ion transporters, such as salinomycin and monesin, which will not be discussed here since they have been recently reviewed in detail [30]. 

## 3. Artificial Ion Channels and Transporters as Therapeutic Agents

Self-assembly can be a powerful tool to use relatively simple (bio)molecules that organize into supramolecular ion channels for membrane insertion to mimic proteins [91]. There are diverse types of artificial ion channels based on supramolecular chemistry that can be grouped in cages and capsules, stacks of macrocycles, nanotubes, and helical structures [92,93]. The concept of self-assembly into chiral supramolecular structures, such as nanotubes, has attracted great attention for the inherent properties of molecular recognition [94], which are at the basis of medicinal chemistry to develop drugs. 

The majority of artificial ionophores are designed for selective ion transport, and they can be divided in cationic and anionic transporters or channels [95]. Anion carriers are the most studied class, and readers interested in an overview of their biological use are referred to an excellent recent review [96]. Readers interested in the chemical details of non-covalent interactions pertaining the design of anion channels, especially for chloride anion transport to address CF, are referred to existing tutorial reviews covering aspects of supramolecular medicinal chemistry [97]. A common limitation regards the undesired concomitant proton transport, although recent research efforts successfully addressed this side effect [98]. The careful insertion of positive charges along the channel interior, for instance, ensures that only anions are transported [99].

While the majority of supramolecular anion transporters target chloride, bicarbonate is attracting interest for therapeutic applications too [100]. Iodide is also selectively transported [101]. Finally, macrocycles have been devised as ion-pair receptors for the concomitant transport of opposite-charged ions, but this topic will not be covered here since it has been comprehensively covered in detailed and excellent recent reviews [102,103]. 

Several biomolecule classes have inspired the design of artificial ion channels. For instance, cyclodextrins have attracted great interest for their biocompatibility [104]. Rhodopsin has inspired the design of supramolecular Cl^−^ channels that can be regulated by visible light, offering means to potentially control the circadian clock [105]. The field of light-activated ion channels is making progress. As an example, Cl^−^ transporters were recently designed to respond to red and blue light to control the transmembrane ion-transport rate [106]. More traditional systems rely on UV-light triggers to enable cis–trans conformational switches [107]. 

Light is not the only stimulus that has attracted interest to regulate ionophores’ activity. Metal–organic supramolecular Cl^−^ channels were designed to be switched off upon ligand binding [108]. Voltage-dependent H^+^/Cl^−^ symport, without uniport activity, was envisaged for new channel-design strategies [109]. Biomimetic approaches have recently produced dual-responsive ion channels that can be controlled by voltage and ligand binding [110], although the research in this area is for the majority still at a fundamental level. Below we discuss more in-detail recent examples of artificial channels and transporters designed for biological applications, mainly as anticancer or AM agents, and that were tested on cells or animal models.

### 3.1. Artificial Anion Channels as Anticancer Agents

**Figure 4:**
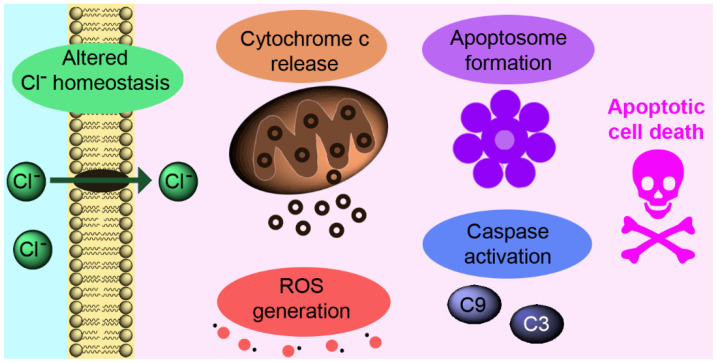
Schematic representation of caspase-mediated apoptosis.

Talukdar’s research group has been very active on the development of artificial ion channels. Bis-diols were reported to self-assemble into amphipathic nanotubes via hydrogen bonds [111]. The diols were tethered at two alkyne ends of a central rigid 1,3-diethynylbenzene moiety and functionalized with dialkylamino groups with different lengths of the alkyl chain to modulate the lipophilicity of the systems. Among the members of the family, compound **C1** (Table 2) had the optimal lipophilicity to allow its translocation across the phospholipid membrane of egg-yolk phosphatidylcholine large unilamellar vesicles. A detailed study allowed to define the selectivity towards Cl^−^ over other anions, such as Br^−^, ClO_4_^−^, F^−^, NO_3_^−^, and I^−^, and the preferred transport mechanism as a Cl^−^/NO_3_^−^ antiport. The Cl^−^ transport ability across cell membranes was also successfully tested, by monitoring the dose-dependent fluorescent quenching of the cell-permeable Cl^−^-selective dye *N*-(ethoxycarbonylmethyl)-6-methoxyquinolinium bromide (MQAE) after incubation of HeLa cells with **C1** for 24 h. The impact of Cl^−^ transport mediated by **C1** on cell viability was studied by MTT assay on different cell lines. The increased Cl^−^ level inside the cells resulted in an increase in cell death. The disruption of Cl^−^ homeostasis caused a change in the mitochondrial membrane potential that was accompanied by an increase in ROS production and release of cytochrome c. A detailed study of the cell death mechanism demonstrated that it was caspase-mediated (Figure 4).

The same group reported **C2** (Table 2) forming channels for Cl^−^ transport [113,114]. **C2** could be obtained upon activation of a precursor, mediated either by an esterase or by glutathione (GSH). The latter is particularly appealing for two reasons. Firstly, GSH is an antioxidant that helps preventing oxidative stress linked to apoptosis in cancer cells [117]. Secondly, high levels of GSH in cancer cells can cause resistance to various chemotherapeutics [118]. The nanotube formed similarly to **C1** through non-covalent interactions. The preferred transport mechanism was a M^+^/Cl^−^ symport, and the channel formation was demonstrated by measuring the ionic conductance across planar lipid bilayer membrane [119] and molecular modelling. The study of cell viability demonstrated that the precursor was more toxic (IC_50_ = 0.5–1.0 μM) than **C2** (IC_50_ = 50 μM). This difference was attributed to the presence of the sulfonyl group that increases cell membrane permeability. Fluorescence measurements demonstrated that cancer cells treated with the precursor had high levels of GSH that effectively produced **C2**. Cytotoxicity studies in the presence of Cl^−^ demonstrated that cell death was connected to Cl^−^ transport, with an effect also on the symport transport of Na^+^ and K^+^. Similarly to **C1**, also in this case the apoptotic pathway was caspase-mediated. Interestingly, the authors showed that 3D cultures of breast cancer cells treated with the **C2** precursor displayed similar effects to those treated with doxorubicin, a potent inhibitor of their growth [120].

A similar compound (**C3**, Table 2) formed a channel capable of M^+^/Cl^−^ symport across unilamellar vesicles and cancer-cell membranes, thus leading to apoptosis. The intrinsic fluorescence of **C3** allowed to live-image cancer cells, to reveal the ability of the system to permeate the cytosol. Real-time analysis showed that the permeation occurred in a few seconds, and in a few minutes the cell morphology changed, and the cell volumes were decreased. Apoptosis was confirmed by mitochondrial membrane depolarization, reactive oxygen species generation with consequent cytochrome c release, activation of the caspase 9 pathway, poly (ADP-ribose) polymerase cleavage, and staining of nuclear contents with propidium iodide [115].

Another example of a Cl^−^-selective transmembrane channel was reported by Zeng and co-workers [116]. The authors described a family of receptors containing an amino acid-based scaffold functionalized with a Cl^−^-binding group. These receptors were able to self-assemble and stack by means of halogen and hydrogen bonding. In particular, the alanine-substituted **C4** (Table 2) showed a good capability for OH^−^/Cl^−^ antiport. **C4** inhibited breast-cancer cell growth with an ic_50_ value of 20 μm, which was lower than that of the anticancer drug cisplatin (IC_50_ = 37 μm) [121].

### 3.2. Artificial Anion Channels as Antimicrobial (AM) Agents

**Scheme 1:**
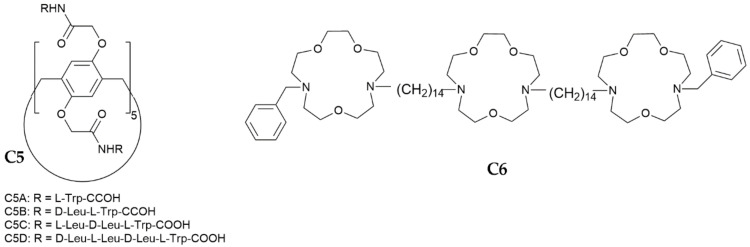
Chemical structures of artificial K^+^ channels recently developed as AM agents [122,126].

Synthetic transmembrane channels have found applications also as AM agents, thanks to their ability to interact with the membrane of prokaryotic cells, similarly to certain antibiotics and AMPs. Hou and co-workers described the ability of tubular pillar [5]arene macrocycles (**C5A–D**, Scheme 1) functionalized with amino acidic sidechains to form channels and insert into the lipid bilayers of Gram-positive bacteria [122]. All the channels were selective for K^+^. **C5B–D** showed AM activity on Gram-positive *S. aureus* that was higher than that of gramicidin A, with **C5B** exhibiting the highest activity (IC_50_ = 0.3 μM). However, in mammalian-cell membrane models, **C5A** showed a weaker insertion ability than **C5B–D**, suggesting that the length of the sidechain played a role in the interaction with membrane lipids. In Gram-positive bacteria *S. epidermidis*, red-fluorescent derivatives could be imaged as rings in the bacterial wall. The authors hypothesized the formation of H-bonds and dipole interactions between the indole residues of the Trp and the membrane lipids, in contrast with the classic electrostatic interactions between positively charged AMPs and negatively charged membrane [123,124]. Conversely, when **C5A–D** were incubated with erythrocytes, the red fluorescence rings were not observed only for **C5A**, with very low hemolytic activity (HC_50_ > 100 μM).

An example of ionophores as AMs and as adjuvants for FDA-approved antibiotics has been reported by Gokel, Patel and co-workers [125]. In previous works, they had described the ability of the crown-ether amphiphile **C6** (Scheme 1) to form ion-conducting pores in liposomal membranes and to improve the antibiotic activity of erythromycin, kanamycin, rifampicin, and tetracycline against Gram-negative bacteria (*E. coli* and *P. aeruginosa*), probably by enhancing the permeability of the membrane [126,127]. Recently, [125], they demonstrated that **C6** can improve the AM activity of tetracycline with ciprofloxacin (i.e., norfloxacin), towards antibiotic-resistant *K. pneumoniae*. The effect was ascribed to a combination of **C6** K^+^ channels abilities to inhibit bacterial growth and to enhance the bacterial membrane permeability that inhibited the efflux pump, a classic mechanism used by Gram-negative bacteria for pumping out AMs.

### 3.3. Artificial Anion Transporters as Anticancer Agents

Gale, Davis, and Sheppard have recently showed that some anionophores can be active in cells [128]. They previously presented an assay employing the genetically-encoded halide-sensitive yellow fluorescent protein (YFP) with encouraging results for several anionophores. They reported an implementation of the YFP-based assay using a standard plate reader. The assay was applied to a set of 22 anionophores including several compound classes, such as *trans*-decalins, substituted cyclohexanes, and the more ideal candidates squaramides, anthracenes, ureas, and thioureas. The data indicated that compounds **T2–T4** exhibited levels of activity comparable to the previously reported **T1** [129]. Hence, the four compounds (Table 3) were selected for further studies.

**T1–T4** mediated anion transport in CF cells (using CF bronchial epithelial cell line, YFP-CSBE), with **T1** and **T2** eliciting a similar response, and **T4** being the least efficient. Cell treatment with purinergic G protein-coupled (p2y) receptor agonist UTP (uridine triphosphate) elevated the intracellular Ca^2+^ levels, thus activating Ca^2+^-dependent Cl^−^ channels, which in turn did not affect the anion transport mediated by **T1–T3**. Conversely, it enhanced **T4**-mediated ion transport. Finally, XTT assays in YFP-CSBE cells revealed the cytotoxicity of **T2**, **T3**, and especially **T4**.

Soto-Cerrato, Pérez Tomàs et al. analyzed in detail the cellular and molecular mechanisms of action of two marine alkaloids (i.e., tambjamines), bearing aromatic enamine moieties **T5** and **T6** (Table 3) [130]. Both promoted transmembrane Cl^−^ and HCO_3_^−^ transport in liposomes. MTT assays on lung cancer epithelial cells A549 revealed a 50% decrease in cell viability within 24 h. It was demonstrated that the IC_50_ for these two compounds was much lower than that observed for the anticancer drug cisplatin (CDDP), i.e., 3.38 ± 0.98 and 1.67 ± 0.29 μM, respectively, vs. 200 μM for CDDP. Furthermore, lysosomal pH modifications in the same cell lines were evaluated using acridine orange, a pH-dependent dye. By treating cancer cells with **T5** and **T6** for 1 h at IC_50_ values, the orange fluorescence in vesicle compartments disappeared, indicating an increase in pH of these organelles and lysosomal alkalization. This may be ascribed to a lysosomal membrane permeabilization induced by **T5** and **T6** that could inactivate its hydrolytic enzymes, thus blocking autophagy. Overall, cell death was caused by a combination of caspase-mediated apoptosis, mitochondrial dysfunctions, and lysosome deacidification promoted by a disruption of the cell homeostasis, all triggered by **T5** and **T6**.

Talukdar et al. conducted transmembrane ion transport studies over a set of bis(sulfonamides) derivatives [131]. The anion binding studies and model, along with the anion transport activity (Cl^−^/NO_3_^−^ antiport mechanism) studies, suggested that **T7** (Table 3) was the most efficient transporter thanks to appropriate lipophilicity and strong anion-binding ability. Cancer-cell and normal-cell viabilities were tested to evaluate how the influx of Cl^−^ into the cell can induce apoptosis. **T7** led to higher cancer cell death relative to the untreated controls, whereas no cytotoxicity was observed in non-cancerous cell lines. Moreover, the correlation between the increased extracellular Cl^−^ levels and caspase-mediated cell death was demonstrated. This pattern is well known to trigger apoptosis [136,137,138].

Shin, Gale, Sessler et al. reported a family of squaramides able to cause a malfunctioning of the ion homeostasis and then induce cell death [139]. Compounds **T8–T10** (Table 3) were the most active Cl^−^ transporters (**T8 > T9 > T10)** in liposomes. The experimental evidence highlighted that Cl^−^ was transported via antiport mechanism (Cl^−^/NO_3_^−^, Cl^−^/HCO_3_^−^, Cl^−^/SO_4_^2−^, or Cl^−^/OH^−^), along with a symport mechanism Cl^−^/H^+^. Fischer rat thyroid epithelial cells (FRT) were incubated with each compound and the Cl^−^ transport activity was found to be similar to that observed in liposomes. MTT assays on HeLa and A549 cancer cell lines revealed an IC_50_ in the range 2–6 μM for **T8–T10**. The apoptotic effect of **T8–T10** was compared against carbonyl cyanide-4-(trifluoromethoxy)phenylhydrazone (FCCP), which is an apoptosis inducer that depolarizes mitochondrial membranes. Cell death was found to be caspase-mediated, rather than necrosis-promoted. The cell death mechanism was further elucidated in another contribution from the same authors [132]. **T8** and **T9** were studied by Kroemer, Zamzami et al. too, on a CFBE cell line expressing the most frequent *CFTR* mutation [140]. Cell treatment for 24 h with **T8** or **T9** revealed they inhibited the autophagic flux, which would have a negative effect on the disease [141,142], thus rendering them more suitable as anticancer agents [142].

Another interesting strategy was presented by Zhang et al. who have successfully constructed an ATP-regulated ion transporter nanosystem for homeostatic perturbation therapy (HTP) and sensitized photodynamic therapy (PDT) [143]. The smart nanotransporter SQU@PCN (porphyrinic porous coordination network incorporated with squaramide **T8**), accumulated in tumor sites avoiding metabolic clearance and side effects. The affinity of phosphates towards metal ions is well known [144], thus the interaction between the nanosystem and ATP was studied. The decomposition of the nanosystem along with the release of **T8** inside cells was observed. **T8** triggered Cl^−^ transport across the cell membrane, increasing the intracellular ion concentration, which disrupted ion homeostasis and further induced tumor cell apoptosis.

**Figure 5:**
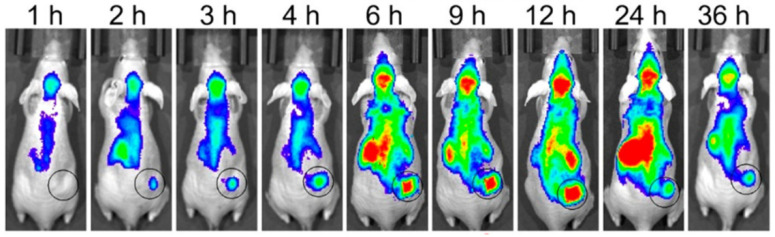
Fluorescence imaging in vivo after intravenous injection with SQU@PCN. Reproduced from ref. [143]. Copyright © 2019 American Chemical Society.

The viability of HeLa cells after SQU@PCN treatment was assessed via MTT assay. The high cytotoxicity (IC50 = 1.36 mg L^−1^) was attributed to the release of **T8** inside cells, suggesting the selectivity of ATP-SQU@PCN interaction in HeLa cancer cells. Irradiation with a 660-nm laser further enhanced cytotoxicity and lowered the IC_50_, suggesting the synergic effect of HPT and PDT in killing tumor cells. The excellent cancer-cell toxicity in vitro was confirmed in vivo. In particular, after 12 h of SQU@PCN intravenous injection, fluorescence intensity reached the maximum, suggesting the accumulation of SQU@PCN, especially in the tumor region (Figure 5). Hence, when mice were subjected to light irradiation at 660 nm for 8 min after 12 h of the SQU@PCN injection, the tumor growth was suppressed, confirming what already observed in vitro [143].

### 3.4. Artificial Cation Transporters as Anticancer Agents

Yang et al. reported a family of synthetic K^+^ transporters [135]. They found that **T11** (Table 3), an α-aminoxy acid derivative, exhibited the greatest K^+^ transport ability at the concentration of 10 μM, through a 1:1 carrier mechanism. Anionic **T11** was able to bind K^+^ through electrostatic interaction and ion coordination by the aminoxy oxygen atom and the two carbonyl groups. When **T11** entered liposomes with pH~6.8, it could be protonated to release K^+^ and freely diffuse through the membrane to complete the carrier cycle. **T11** was found to be extremely selective towards K^+^ over other alkaline metal ions with no Cl^−^ transport across membranes. However, by conducting the HPTS assay in the presence of valinomycin and FCCP, the electrogenic transport mediated by **T11** displayed H^+^ > K^+^, suggesting how **T11** could promote the transport of H^+^ and K^+^ independently.

**T11** was also tested on human ovarian cancer HEYA8 cells, and no ion transport across the plasma membrane was detected. The authors hypothesized that the pH gradient in the intermembrane space (IMS) could be a driving force for the transport of K^+^ and H^+^. **T11** could freely move through the IMS, in which **T11** is present in its anionic form and entraps K^+^. The exchange K^+^/H^+^ finally concluded the cycle. This hypothesis was then confirmed in HeLa cells and in human ovarian SKOV3 cells. The authors further evaluated the mitochondrial ROS production, respiration, and mitochondrial morphology in HEYA8 cells. It was demonstrated that the transport mediated by **T11** caused the damage of the mitochondrial functions, due to ion homeostasis dysfunction. Moreover, **T11** mediated K+ transport also in ovarian cancer stem cells (CSCs) and it inhibited their growth at 5 μM. This behavior was not observed in the other cancerous and not-cancerous cell lines tested. The high selectivity towards CSCs prompted the authors to evaluate the effect of **T11** on tumor growth in nude mice. CSCs were thus incubated ex vivo with **T11** or paclitaxel (PTX) and then re-injected into nude mice after 10 days, to reveal a significantly decreased ability to form tumors, relative to controls [135].

Zeng et al. reported the novel class of cation transporters **T12–T14** (Table 2), which were characterized by three modular components (i.e., a headgroup, a flexible alkyl-chain derived body, and a crown-ether derived foot for anion binding) [133]. The selective and efficient transport of K^+^ ions across large unilamellar vesicles promoted by **T12–T14** was demonstrated, as suggested by the EC_50_ = 0.18–0.41 mol % relative to the lipid. Moreover, the most active transporter displayed a potent anticancer activity with low values of IC_50_ towards HeLa and prostate cancer cells PC3.

### 3.5. Artificial Ion Transporters as Antimicrobial (AM) Agents

**Scheme 2:**
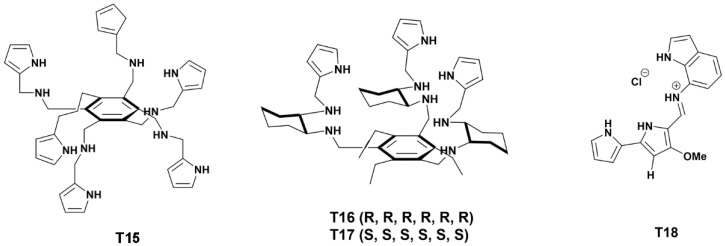
Chemical structures of **T15–T18** [145].

Quesada et al. used 6-indol-7-yl-decorated tambjamine-like compounds **T15–T18** (Scheme 2) against Gram-positive and Gram-negative bacterial strains, as well as clinical isolates [145]. In particular, only **T18** inhibited the growth of Gram-negatives, and it was also the most potent anionophore of the series, with good hemocompatibility.

## 4. Conclusions and Future Perspectives

**Figure 6:**
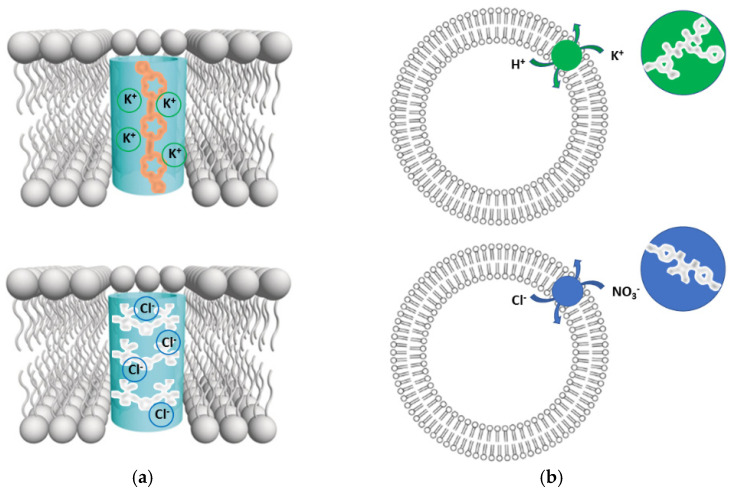
Examples of artificial ion (**a**) channels and (**b**) transporters for membrane insertion that derive from supramolecular-chemistry design.

In this Review, we focused on the latest developments in the design of ion channels and transporters for therapeutic use. Firstly, we mentioned a few well-known natural examples based on biomolecules that have inspired biomimetic design, and then we have analyzed more in detail the case of synthetic ionophores (Figure 6) that were tested on biological models, including cells and animals.

There are many challenges to overcome, to bring artificial ionophores to the clinic. Besides biocompatibility and selectivity for the target cells, ion selectivity is key. This is not always straightforward, for instance for cation transport. However, good K^+^ selectivity over Na^+^ can be attained [146,147,148]. Interestingly, simple structural changes can reverse the selectivity and favor Na+ transport [149].

Sometimes the ionophore performance can be improved through the combination of different therapeutic agents or functional molecular components. For instance, addition of biomolecules, such as the K^+^ transporter valinomycin, to an anion transporter proved to be an effective strategy [150]. Combination with responsive polymers is another approach that is being explored to gain control over transport [151]. Moreover, external stimuli are very attractive to modulate the gating mechanisms, and amongst them, light is the new frontier for therapeutic approaches, especially to control neuronal ion channels [6].

Besides ion channels, also many other types of transmembrane channels offer unexplored therapeutic opportunities. Aquaporins selectively transport water and are impaired in several water balance disorders, such as nephrogenic diabetes insipidus [152]. Biomimetic approaches to develop artificial water channels capable of excluding ions and protons is indeed another area of great innovation potential [153,154]. New characterization methods to assess the transport across membranes are continuously being developed and are envisaged to assist research towards new therapies [155]. Furthermore, they could serve also for diagnosis and consequent development of personalized therapies, as demonstrated for the intestinal current measurements in the case of CF [156]. Overall, as we advance our understanding of artificial ion channels’ structure-activity relationship, and of the biochemical pathways involving ion transport in physiological and pathological contexts, we will witness a bright future at the interface between supramolecular chemistry and biomimetic design to innovate therapy.

## Figures and Tables

**Table 1:** Ion-channel blockers and chelators that are drug candidates in clinical trials against cancer. The table includes drugs that are being considered or in clinical trials as anti-metastatic agents, which may counteract the ionic imbalance in disseminating or disseminated cancer cells. Adapted from [52] under a Creative Commons license.

Ion	Function	Drug Candidate	Feature	Target Cancer	Phase
Ca^2+^	Blocker	Amlodipine besylate	Selective for L-type Ca^2+^ channels, antihypertensive drug	Metastatic triple negative breast cancer	1,2
	Blocker	Verapamil	Antihypertensive drug	Brain Cancer	2
K^+^	Blocker	Imipramine	Targets voltage-gated channels, drug against depression	HER2 Positive Breast Carcinoma	0
Cu^2+^	Chelator	Trientine	Anti-angiogenesis, normally used to treat Wilson disease	Fallopian Tube Cancer Ovarian Neoplasms Malignant/primary peritoneal cancer	1,2
		Salicylaldehyde pyrazole hydrazone	Anti-angiogenesis	-	-
		Tetrathiomolybdate	Drug used against primary biliary cholangitis, Wilson Disease	Prostate cancer, carcinoma, colorectal cancer non-small cell lung cancer	1,2
		Penicillamine	Drug against cystine renal calculi	Brain and CNS tumors	2
		Disulfiram	Drug against alcohol dependency	Metastatic breast cancer Metastatic pancreatic cancer	2
		Clioquinol	Drug against dermatitis and eczema	Acute lymphocytic leukemia Acute myeloid leukemia Chronic lymphocytic leukemia	1 *
Fe^2+^/Fe^3+^	Chelator	Ciclopirox olamine	Drug against onychomycosis, foot dermatoses	Hematologic malignancy, acute lymphocytic leukemia, advanced solid tumors	1
		Thiosemicarbazones	Drug against renal failure, renal artery stenosis	Unspecified adult solid tumor, protocol specific, prostate cancer/metastatic well differentiated neuroendocrine neoplasm	1
		Deferiprone	Drug against cardiomyopathy, iron overload, deteriorating renal function	Colon cancer, breast cancer, rectal cancer, urethral carcinoma	2
		Deferasirox	It suppresses N-Cadherin; drug against acute undifferentiated leukemia/ iron overload	Breast cancer, leukemia	2 *
		desferrioxamine	restores E-Cadherin localization. Drug against cardiomyopathy/iron overload	Acute myeloid leukemia/acute lymphoblastic leukemia/ myelodysplastic syndrome	*
Many	Blocker	Chlorotoxin	NKCC channel blocker	Breast cancer/non-small cell lung cancer/melanoma/ brain neoplasm	1,2
Na^+^	Blocker	Propranolol	Targets VGSC ^1^, used for post-traumatic stress disorder, brain injuries	Invasive epithelial ovarian cancer, primary peritoneal carcinoma, fallopian tube cancer, cervical cancer, pediatric cancer/breast cancer	1,2
		Ranolazine	Targets VGSC ^1^, used for pulmonary hypertension, angina	Adenocarcinoma of the prostate, bone metastases, soft tissue metastases	-
		Phenytoin	Targets VGSC ^1^, used for acute kidney injury/impaired renal function/kidney failure	Pancreatic cancer, locally advanced breast cancer and large operable breast cancer/metastatic breast cancer, metastatic pancreatic cancer	2,3
		Carbamazepine	Targets VGSC ^1^, used for bipolar disorder (bd), epilepsy, erythromelalgia	Brain and central nervous system tumors, glioblastoma	1,2
		Valproate	Targets VGSC ^1^, used for acute kidney injury/impaired renal function/kidney failure	Advanced cancer/prostate cancer, breast cancer, pancreatic cancer	1,2
		Lamotrigine	Targets VGSC ^1^, used for bipolar disorder	Brain and central nervous system tumors/malignant glioma	2,4
		Ranolazine	Targets VGSC ^1^	Adenocarcinoma of the prostate, bone metastases, soft tissue metastases	-
		Ropivacaine	Targets VGSC ^1^, used for anesthesia, conduction/ arthroplasty, replacement/ postoperative pain	Malignant neoplasm of breast	3
		Lidocaine	Targets VGSC ^1^, used for anesthesia	Lung cancers, unspecified adult solid tumor, prostate cancer	1,2
		Riluzole	Targts VGSC ^1^	Breast cancer/metastatic cancer	1 *

* Denotes terminated clinical trials. ^1^ VGSC = voltage gated sodium channels.

**Table 2:** Cl^−^ selective artificial channels recently developed to induce apoptosis as anticancer agents.

Compound	LogP	EC50 (μM)	N	Cell Line	Ref.
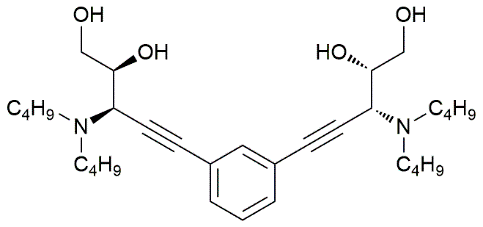 **C1**	5.58	2.7 ± 0.1	2.4 ± 0.1	HeLa, MCF-7, U2OS	[112]
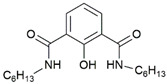 **C2**	5.54	6.5 ± 0.3	2.4 ± 0.2	MCF-7	[113,114]
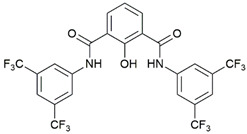 **C3**	7.30	0.47	1.79	MCF-7	[115]
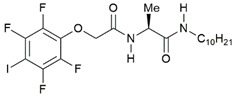 **C4**	-	2.37	3.5	BT-474	[116]

**Table 3:** Artificial ion transporters that were recently developed as anticancer agents. **T1–T10** are selective for Cl^−^ transport, while **T11** and **T12** for K^+^.

Compound	LogP	EC_50_	*n*	Cell Line	Ref.
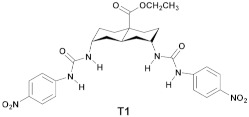	4.5	-	-	YFP-CSBE	[129]
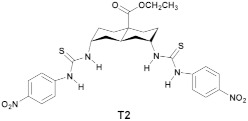	4.8	-	-	YFP-CSBE	[128]
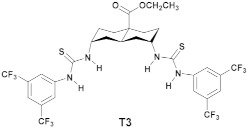	9.3	-	-	YFP-CSBE	[128]
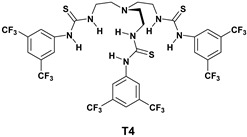	10.4	-	-	YFP-CSBE	[128]
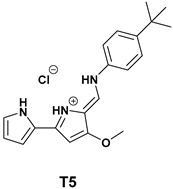	4.13	50 ± 8 nM	1.14 ± 0.3	A549	[130]
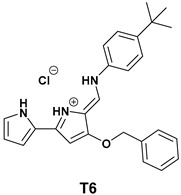	5.58	60 ± 3 nM	1.20 ± 0.09	A549	[130]
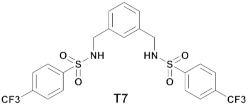	4.84	5.89 ± 0.15 μM	-	MCF-7, U2OS, A549, NIH3T3	[131]
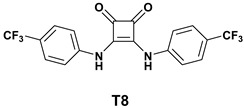	3.87	1.5 nM	1.2(0.09)	HeLa, A549	[132]
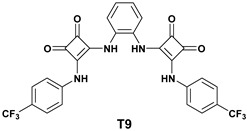	-	2.12 nM	1.22 (0.05)	HeLa, A549	[132]
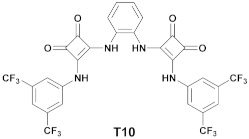	-	4.1 nM	0.7 (0.2)	HeLa, A549	[132]
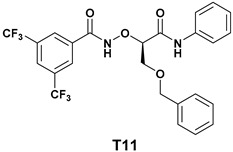	-	4.04 mol %	0.84	HeLa, HEYA8, SKOV3, CSCs	[133]
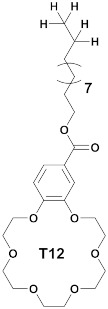	-	0.22 ± 0.02 μM	-	PC3	[134]
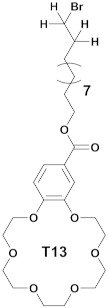	-	0.17 ± 0.01 μM	-	PC3	[135]
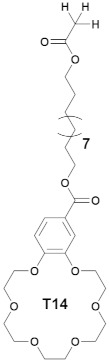	-	0.15 ± 0.01 μM	-	PC3	[135]

## Data Availability

Not applicable.
